# Pre-Procedural Statin Use Is Associated with Improved Long-Term Survival and Reduced Major Cardiovascular Events in Patients Undergoing Carotid Artery Stenting: A Retrospective Study

**DOI:** 10.3390/jcm7090286

**Published:** 2018-09-17

**Authors:** GianLuca Colussi, Francesca Zuttion, Bruno Bais, Pierluigi Dolso, Mariarosaria Valente, Gian Luigi Gigli, Daniele Gasparini, Massimo Sponza, Cristiana Catena, Leonardo A. Sechi, Alessandro Cavarape

**Affiliations:** 1 Division of Internal Medicine, Department of Medicine, University of Udine, 33100 Udine, Italy; fiammetta.zutt@gmail.com (F.Z.); bruno.bais@asuiud.sanita.fvg.it (B.B.); cristiana.catena@uniud.it (C.C.); leonardo.sechi@uniud.it (L.A.S.); alessandro.cavarape@uniud.it (A.C.); 2 Division of Neurology, Department of Medicine, University of Udine, 33100 Udine, Italy; pierluigi.dolso@asuiud.sanita.fvg.it (P.D.); mariarosaria.valente@uniud.it (M.V.); gianluigi.gigli@uniud.it (G.L.G.); 3 Division of Interventional Radiology, Department of Radiology, Academic Hospital of Udine, 33100 Udine, Italy; ozoudine@gmail.com (D.G.); massimo.sponza@asuiud.sanita.fvg.it (M.S.)

**Keywords:** statins, atherosclerosis, carotid artery stenting, mortality, cardiovascular event

## Abstract

Carotid artery stenting (CAS) is a minimal invasive procedure used to resolve carotid occlusion that can be affected by peri-procedural complications. Statin use before CAS has shown to reduce peri-procedural risk and improve survival, though time-dependent cofactors that influence mortality has not been considered. The aim of this study was to evaluate long-term survival of patients who undergo CAS considering new occurred major adverse cardiovascular event (MACE) as time-dependent cofactor. In this study, 171 high cardiovascular risk patients (age 72 ± 8 years, 125 males) were enrolled after CAS procedure and were followed for a median of 8.4 years. Death occurred in 44% of patients with a mean time to death of 69 ± 39 months and MACE in 34% with a mean time of 35 ± 42 months. In patients who used or not statins at baseline, death occurred in 33% and 65%, respectively (*p* < 0.001). Survival analysis showed that statin use reduced risk of death (hazard ratio HR 0.36, 95% confidence interval CI 0.23–0.58, *p* < 0.0001). Including MACE as time-dependent variable did not change beneficial effects of statins. Additionally, statin use was associated with a protective effect on MACE (HR 0.48, 95% CI 0.27–0.85, *p* = 0.012); particularly, the prevalence of stroke was reduced by 59% (*p* = 0.018). In multivariate analysis, effects of statins were independent of demographic and anthropometric variables, prevalence of cardiovascular risk factors, renal function, antiplatelet use, and MACE occurrence. In conclusion, use of statins before CAS procedure is associated with increased long-term survival and reduced MACE occurrence. This evidence supports the hypothesis that statin use before CAS might be beneficial in high risk patients.

## 1. Introduction

Statins are effective in cardiovascular primary and secondary prevention by reducing the incidence of cardiovascular events and mortality [1]. Statins reduce total and LDL-cholesterol levels that are implicated in the development and progression of atherosclerotic arterial lesions with an effect that is wider than effects related just to LDL-cholesterol reduction [2]. Although evidence of the protective effects of statins in cardiac and extra-cardiac revascularization is recognized [3,4], benefit of statins use in the prevention of major adverse cardiovascular events (MACE) and long-term mortality after CAS has been suggested in a single observational study [5]. In this multicenter Italian study, more than 1000 patients were recruited after primary CAS, and statins were taken by 43% of patients before the procedure. After a follow-up of 5 years, patients who took statins before CAS had lower mortality and incidence of perioperative stroke than patients who did not take statins. The better survival was imputed mainly to a lower incidence of perioperative stroke and late-occurred ischemic stroke [5]. However, this latter evidence was only borderline significant, and the study did not consider time-dependent events that could have influenced mortality, thus misestimating the potential beneficial effect of statins on different outcomes. Similar results on post-operative mortality were shown in the recent large retrospective analysis of Rizwan et al. on data of the Premier Healthcare Database, a large American database that includes data of more than 700 U.S. hospitals [6].

Carotid artery disease as represented by atherosclerotic plaques is a common subclinical organ damage seen in patients with cardiovascular risk factors such as diabetes, hypertension, dyslipidemia, and smoking history and its prevalence increases progressively according to age. Atherosclerotic plaque progression in internal carotid artery over a significant percentage of lumen stenosis is associated with high risk of cerebrovascular events such as transient ischemic attack (TIA), ischemic stroke, and retinal artery occlusion which can permanently impair patient’s autonomy and life-expectancy. Carotid artery stenting (CAS) is an effective procedure for patients with critical or symptomatic internal carotid artery stenosis for preventing cerebrovascular events and mortality [7]. This procedure might be preferred in high risk patients because it is less invasive and associated with lower peri-procedural cardiac events than surgical endarterectomy [8]. However, CAS is affected by an increased risk of post-procedural stroke respect to endarterectomy and the best choice between the two procedures is left just to local experience [9].

Recently, prospective cohort studies showed that pre-procedural use of statins can reduce the risk of peri-procedural CAS complications including stroke and mortality, but so far data on very long-term survival considering time-dependent covariates that can influence survival beyond baseline conditions have not been reported [10,11,12]. In the study of Gröschel et al. conducted in Germany, 180 patients were followed prospectively 30 days after CAS for high-grade symptomatic carotid stenosis. Patients who took statins before procedure (29% of all patients) had a lower incidence of the composite endpoint (stroke, myocardial infarction, and death) than patients who did not take statins; 4% and 15%, respectively [10]. Similar results were shown more recently by Reiff et al. in 344 other patients, 60% of whom took statins before CAS [11]. Also, Hong et al. demonstrated on 397 Korean patients that the protective post-procedural benefit of statins used before CAS on cardiovascular events and death was clearly dose-dependent [12]. Very recently, a meta-analysis on randomized trial and observational studies confirmed a beneficial effect of the pre-procedural statins’ intake on peri-procedural risk of stroke and death [13].

Considering promising results and limitations of previous studies, our aim was to assess very long-term survival (beyond 5 years of follow-up) of patients who undergo CAS procedure and who were taking or not taking statins at baseline in a retrospective analysis that considers the occurrence of new MACE during follow-up as time-dependent covariate.

## 2. Patients and Methods

In this retrospective study we included consecutively patients that underwent primary CAS procedure for a significant carotid artery stenosis after multidisciplinary evaluation by an internist, a neurologist, and an interventional radiologist. When appropriate a vascular surgeon was involved to confirm clinical indication to CAS procedure. All patients underwent CAS between 2003 and 2008 and were followed at our Cardiovascular Risk and Atherosclerotic Prevention Center until the end of 2016. Patients were considered for CAS procedure when they were at elevated surgical risk for age or for the presence of severe comorbidities, refused endarterectomy instead of CAS, had a favorable carotid anatomy. Carotid revascularization was offered to patients with a stenosis of the internal carotid artery confirmed by computer tomography or magnetic resonance angiography greater than 60% of the internal lumen or if they were asymptomatic but presented a confirmed stenosis of more than 70% and showing cerebral imaging of ipsilateral silent infarctions. Symptomatic patients were considered those with history of TIA, stroke or retinal artery occlusion occurred in the six months before evaluation and whose cerebral ischemic lesions were confirmed by radiological imaging that is concordant with the lesion’s side. 

All patients were evaluated by the internist for the presence of major cardiovascular risk factors according to current guidelines [14]. Blood pressure, anthropometric and other general clinical variables were collected, and biochemical laboratory measurements had been performed for assessing lipid and glucose metabolism and renal function. Diagnosis of hypertension was defined by current use of antihypertensive drugs or by at least two in-office independent repeated measurements of systolic and/or diastolic blood pressure levels greater than 139 and 89 mmHg, respectively. Diabetes was defined by use of oral antidiabetic drugs or insulin or by at least two independent morning fasting glucose measurements greater than 125 mg/dL and/or a glycated hemoglobin greater than 6.4%. Dyslipidemia was defined by use of lipid-lowering drugs or at least two independent morning plasma fasting total cholesterol measurements greater than 200 mg/dL. Body mass index was defined as weight in Kg over squared height in meters and glomerular filtration rate has been estimated (eGFR) by the equation of Cockcroft-Gault. Baseline prevalence of cardiovascular diseases such as chronic angina, ischemic cardiomyopathy, cerebrovascular diseases, atrial fibrillation, and peripheral artery diseases was assessed by clinical interviews, medical visits, and patients’ medical records. Dual antiplatelet therapy with aspirin (ASA) 100 mg/day and either clopidogrel 75 mg/day or ticlopidine 500 mg/day was started at least 3 days before CAS procedure in all patients. 

All patients were evaluated by a neurologist for assessment of TIA or stroke signs and by an ophthalmologist in case of retinal artery occlusion. A neurologist or radiologist performed the carotid duplex ultrasound in high risk patients that came into the attention of our Center or the neurological division. Carotid artery stenosis was quantified by the criteria of the North American Symptomatic Carotid Endarterectomy Trial (NASCET). Finally, an interventional radiologist checked clinical indications, evaluated technical aspects from radiological imaging, and confirmed CAS procedure [15].

CAS procedure was performed with the assistance of an anesthetist that induced a mild sedation of patients and monitored they vital signs during the procedure providing advance life support when necessary. After local anesthesia, the interventional radiologist performed stenting via the femoral artery or via brachial or radial arteries when the femoral access was problematic. A temporarily filter device was introduced with catheter to reduce the risk of cerebral embolization during angioplasty and stenting. Intravenous heparin (100 U/Kg) was infused during the procedure. Immediately after CAS procedure patients were taken to the ward of the Department of Medicine to monitor vital parameters and possible signs of acute cerebrovascular complications. Generally, patients were discharged from the hospital on the third day after CAS procedure. Carotid duplex ultrasound was repeated at 1 and 6 months after CAS procedure then at 1 and 2 years from procedure to assess carotid restenosis. Dual antiplatelet therapy was continued for at least 3 months after CAS, then a single antiplatelet drug was routinely taken for chronic use (mainly ASA if tolerated). Patients were revaluated by the internist at 3 and 6 months and then annually to monitor cardiovascular risk factors, adherence to treatments, and register new MACE occurrence. Patients not compliant to regular controls were contacted by telephone and electronic records were consulted when available to check the clinical status or death. MACE that occurred within 1 month from CAS procedure were considered as peri-procedural event. Giving the retrospective design of this study, causes of death where not available for most patients. For definition of MACE, we considered one of the following events: TIA, ischemic or hemorrhagic stroke, myocardial infarction, acute coronary artery disease, or arterial revascularization procedures other than CAS.

This study was approved by the Institutional Review Board of the University of Udine (40/IRB_COLUSSI_18) and the written consent of participants was collected according to the Italian law on retrospective studies. 

### Statistical Analysis

Data are presented as mean ± standard deviation for normal-distributed variables and as median (interquartile range) for skewed ones. Normal distribution of variables was assessed by histogram view and the Shapiro-Wilk normality test. Skew variables were log-transformed before use in parametric analysis. Comparisons between means were performed with the Student-*t-*test, whereas comparisons between proportions with chi-square or exact Fischer tests as needed. Multivariate analysis of dichotomous dependent variables was performed with the logistic regression and coefficients were reported as adjusted Odds Ratio (OR) with the 95% confidence interval (CI).

**Figure 1:**
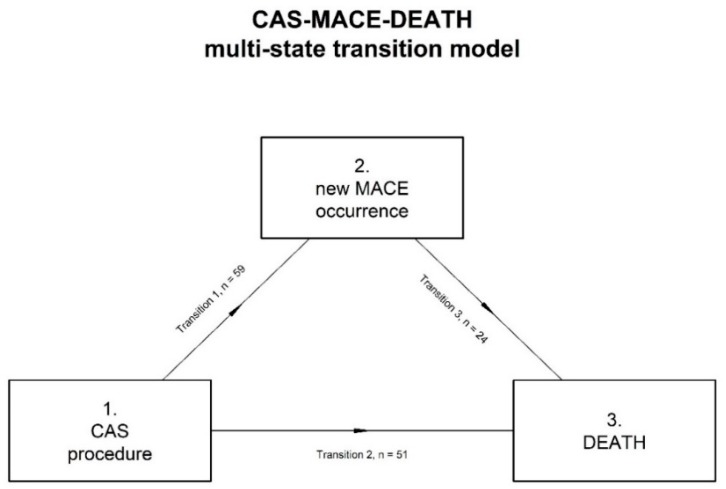
Multi-state transition model (illness-death) used to analyze sample data. Arrows express the transition from one state to another. Transitions are numbered from 1 to 3 and the number of patients in each transition is indicated.

**Figure 2:**
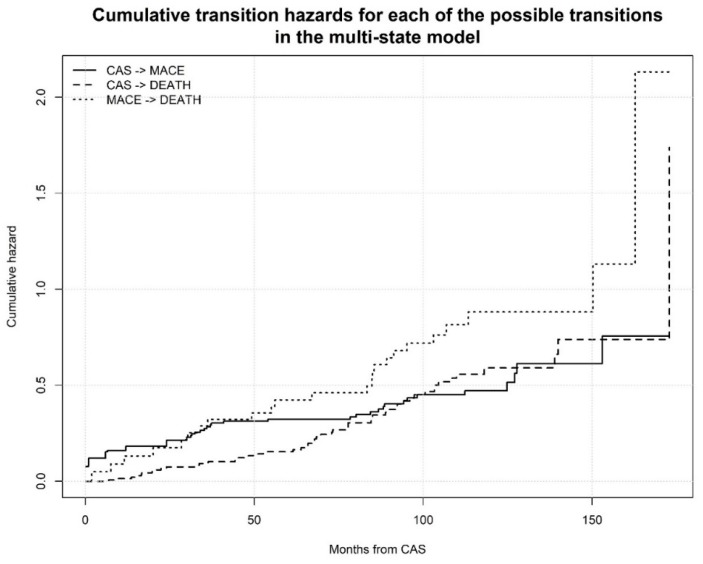
Cumulative transition hazards for each of the possible transition in the multi-state model. Proportional hazards assumption appears clear.

For the survival analysis we consider both a classic Cox model with time to MACE as a covariate and a multi-state transition (illness-death) model with the occurrence of new MACE during follow-up as the only time-dependent covariate (Figure 1) [16]. Survival analysis of patients stratified in statin users and non-users was represented by Kaplan-Meier curves and analyzed by the not-parametric log-rank test. Influence of covariates including statins use on survival rate was assessed by Cox proportional hazards analysis considering a Markov model for the multi-state transition with a clock-forward approach [17]. Transition hazards in MACE or death from baseline status were considered to be proportional (Figure 2). In all models, anthropometric variables, major cardiovascular risk factors, renal function as expressed by estimated glomerular filtration rate with the Cockcroft-Gault formula, use of statin and antiplatelet drugs before CAS procedure, and months to first MACE occurrence were included as covariates. Coefficients were reported as Hazard Ratio (HR) and 95% CI. Because all patients with dyslipidemia took statins we did not include dyslipidemia as covariate for collinearity problems. 

For the multi-state Markov model analysis, age, BMI, and renal function were categorized to make clear the interpretation of HR. Age was divided into three classes according to age at enrollment lower than 65 years, between 65–75 years, and greater than 75 years BMI was divided into three classes according to BMI lower than 25 Kg/m^2^, between 25–30 Kg/m^2^, and greater than 30 Kg/m^2^. Renal function was classified into three classes that included renal failure of stage I, stage II, and stage III + IV, respectively. Stages were defined as established by the Kidney Disease: Improving Global Outcomes (KDIGO) criteria on estimated glomerular filtration rate [18]. For statistical reasons, when deaths and MACE overlapped, we arbitrary considered that MACE occurred one week before death. 

Data collection was complete without missing values and the null-hypothesis was rejected when tests’ two-tails probabilities were equal or less than 5%. For statistical analysis and graphs we used R software (Version 3.4.3) with survival (Version 2.41–3), mstate (Version 0.2.10), survminer (Version 0.4.2), and diagram (Version 1.6.4) packages [17,19].

## 3. Results

In this study, 171 patients, age 72 ± 8 years, who underwent CAS procedure were enrolled and followed for a median of 101 months (range 2–173). Death occurred in 44% of patients after a mean follow-up of 69 ± 39 months and a new MACE occurred in 34% after 35 ± 42 months (Table 1). Baseline prevalence of major cardiovascular risk factors in the sample was: male sex 73%, smoking history 67%, hypertension 85%, diabetes 46%, dyslipidemia 71%, and pre-existent MACE 68%. Prevalence of cardiovascular diseases at baseline included: atrial fibrillation 9%, ischemic heart disease 36%, and peripheral artery diseases other than carotid stenosis 33%. Moderate renal failure (eGFR less than 60 mL/min/1.73 m^2^) was observed in 36% of patients. Statins were used by 66% of patients before CAS, and molecules consisted of simvastatin in 41%, atorvastatin in 40%, rosuvastatin in 15%, and pravastatin in 4% of statin users. The range of statin dose taken by patients was 20–40 mg for simvastatin, 20–80 mg for atorvastatin, 10–40 mg for rosuvastatin, and 20–40 mg for pravastatin.

Patients who died within the study period were older at enrollment (+4 years) and had greater prevalence of diabetes (+79%) and atrial fibrillation (3-fold), but lower use of statins (–38%) and a shorter time of follow-up (–32 months) and time to first MACE occurrence (–23 months) than alive/censured patients (Table 1). In patients who developed new MACE, death occurred in 41% after 85 ± 45 months, whereas in patients without new MACE, death occurred in 45% after 86 ± 45 months. Characteristics of patients with MACE did not differ from those of patients who did not have MACE during follow-up (Table 1). Patients that use statins at baseline were 5 years younger at enrollment and had greater eGFR (+13%), more prevalent dyslipidemia and ischemic heart disease, and longer time to death (+9 months), time to new MACE (+26 months), and average follow-up time (+19 months) than patients that did not use statins. Death among statin users was 49% lower than among non-users (Table 1). Peri-procedural complications after CAS occurred in 14 (8.2%) patients, of which 6 (3.5%) with and 8 (4.7%) without baseline statin use (*p* = 0.076). In the multivariate logistic-regression model including sex, age, smoking history, baseline BMI, eGFR, use of antiplatelet drugs, prevalence of diabetes, hypertension, and pre-existent MACE, statin use was negatively associated with peri-procedural complications (adjusted OR 0.16, 95% CI 0.04–0.58, *p* = 0.007). Peri-procedural complications consisted only of TIA/minor stroke. Neither peri-procedural deaths nor other cardiovascular events were reported.

**Figure 3:**
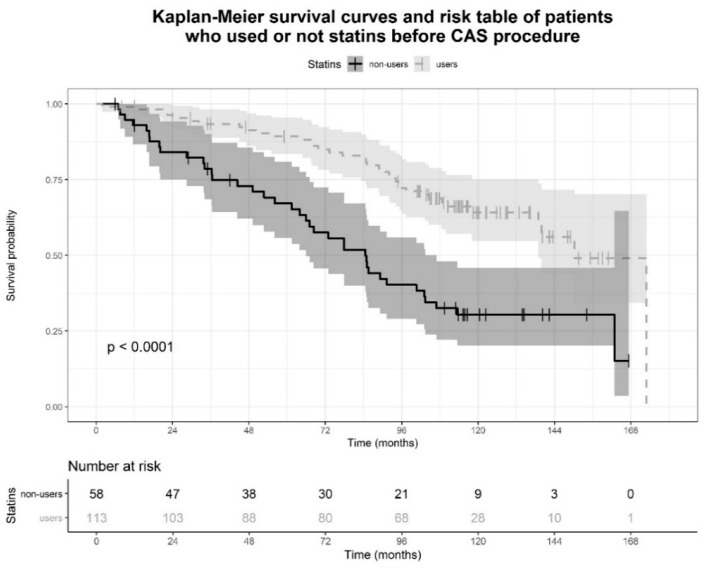
Kaplan-Meier survival curves with 95% confidence intervals and risk table of patients stratified in statin users and not-users. Vertical lines represent censored patients. Probability of the log-rank statistic, *p*, is reported.

Kaplan-Meier curves of patients stratified in statin users and non-users are presented in Figure 3. Patients who used statins pre-procedurally had a better survival rate than patients who did not (unadjusted HR 0.36, 95% CI 0.23–0.58, *p* < 0.0001). In the classic Cox model, the effect of statins on mortality (adjusted HR 0.56, 95% CI 0.33–0.94, *p* = 0.028) was independent of sex, smoking history, baseline BMI, estimated creatinine clearance, use of antiplatelet drugs and prevalence of hypertension, diabetes and preexistent MACE, and time of occurrence of new MACE. Other variables independently associated with mortality were age at enrollment (adjusted HR 1.08, 95% CI 1.04–1.13, *p* < 0.001), baseline prevalence of diabetes (adjusted HR 2.45, 95% CI 1.49–4.04, *p* < 0.001), and months to occurrence of a new MACE (HR 0.987, 95% CI 0.981–0.992, Table 2). 

Analysis of the Markov multi-state model is shown in Table 3. Proportion of patients and mean ± standard deviation values in different class of age, BMI, and stage of renal failure are showed in Table 4. All HR were adjusted for all covariates. The only factor that affected the transition from CAS procedure to MACE (transition 1 in Figure 1) was statin use (HR 0.48, 95% CI 0.27–0.85, *p* = 0.012). Factors that affected transition from CAS to death (transition 2 in Figure 1) were age at enrollment greater than 75 years (HR 4.47, 95% CI 1.23–16.10, ref. age < 65 years, *p* = 0.023), prevalence of diabetes (HR 2.29, 95% CI 1.23–4.27), and statin use (HR 0.43, 95% CI 0.22–0.84). Factors that affected transition from MACE to death (transition 3 in Figure 1) were age at enrollment equal or greater than 65 years (age 65–75 years HR 0.04, 95% CI 0.004–0.345, *p* = 0.004 and age > 75 years HR 0.12, 95% CI 0.02–0.84, *p* = 0.033, ref. age < 65 years), renal failure greater than stage II (HR 46.5, 95% CI 2.09–1034.0, *p* = 0.015, ref. stage I), prevalence of diabetes (HR 5.00, 95% CI 1.26–20.0, *p* = 0.022), and statin use (HR 0.12, 95% CI 0.03–0.47, *p* = 0.002). Analysis of new MACE or death prevalence during follow-up did not change according to different statin types.

In Table 5, types of principal cardiovascular events that occurred during the follow-up were analyzed in patients who used or not statins before CAS. The only difference was seen in the prevalence of fatal and non-fatal stroke (59% lower in patients who used statins, *p* = 0.018). No differences were seen for the prevalence of transitory ischemic attack or fatal and non-fatal ischemic heart disease.

## 4. Discussion

In this retrospective study, we showed that pre-procedural statin use is associated with improved long-term survival in patients who undergo carotid artery angioplasty and stenting. Additionally, statin use was associated with a lower risk of new MACE occurrence after CAS procedure in a multi-state (illness-death) model analysis. These effects of statins were independent of cofactors. 

The protective effect of statins on the onset of peri-procedural complications and long-term cardiovascular events and mortality after angioplasty and stenting has been demonstrated in coronary and other arterial sites [4,20], as well as in carotid surgical endarterectomy [21]. Previous studies have extended this evidence also in carotid artery stenosis regarding peri-procedural complications [6,11,13] and 5-years mortality [5]. Our study confirms previous observations showing a beneficial effect of statins on survival rate and extends this observation over 8 years after CAS procedure also providing additional evidence of beneficial effect of these drugs on new MACE occurrence and, particularly, on the new onset of fatal and non-fatal stroke. Most importantly, our results come from a more accurate multi-state Markov model that takes into account contemporary time-dependent events (new MACE occurrence) that influence mortality rate and that cannot be considered in a classic Cox regression model of survival analysis [16]. 

Although in our study statins effect on mortality was independent of MACE occurrence during follow-up, time to MACE was inversely associated with mortality in the classic Cox model, so that the earlier was MACE occurrence the higher was mortality rate (about 1% per month, Table 3). Additionally, in the Markov model patients younger than 65 years with MACE had a greater risk of death than older patients, although in the classic Cox model age was associated with mortality independently of MACE. This observation shows the higher accuracy of the multi-state model than the classic Cox model to analyze time-to-event studies and suggests that MACE occurrence in younger patients after CAS can be greater than in the older ones.

Angioplasty and stenting are procedures that activates vascular wall inflammation, platelet activation, and induce a local prothrombotic state that is responsible for vessel restenosis or thrombo-embolic events [22]. Also, in the case of CAS elderly patients are at increased risk of peri-procedural complications such as embolic stroke and/or death than patients treated with surgical endarterectomy [23,24]. Nevertheless, CAS is a less invasive intervention than surgical endarterectomy and more appealing for patients at elevated surgical risk such as the elderly and/or patients with important comorbidities. This study suggests that an optimization of drug therapy before CAS might improve procedure effectiveness [25]. In this regard, a randomized controlled trial with high dose rosuvastatin on peri-procedural complications in dyslipidemic patients who undergo CAS procedure is ongoing and will provide important information in the next years [26].

Statins inhibit 3-hydroxy-3-methylglutaryl-CoA-reducatse enzyme that is involved in endogenous cholesterol synthesis in the liver and, by that, main statin effect is to reduce circulatory total and LDL cholesterol levels. The lipid-lowering effect of statins is considered fundamental to stabilize atherosclerotic plaque in which LDL cholesterol exerts an important etiopathogenic role, so that statins reduce cardiovascular risk and mortality [2]. However, Hong et al. had demonstrated a dose-dependent protective effect of statins on CAS peri-procedural complications that is independent of baseline lipid profile suggesting a possible pleiotropic effect of these drugs beyond their lipid effect [12]. Accordingly, a prothrombotic and/or proinflammatory state is associated with subclinical arterial damage in cardiovascular risk patients [27,28] and statins have clearly shown antithrombotic and antinflammatory properties that might contribute to their role in preventing cardiovascular disease and mortality [29]. We hypothesize that such a pleiotropic effect of statins might be involved in early and late protective effects of these drugs on cardiovascular event and mortality after CAS by stabilizing the atherosclerotic plaque. Accordingly, Tadros et al. demonstrated that in patients who undergo CAS, pre-procedural statin use is associated with less embolic debris captured by the carotid filter during the procedure, thus, providing evidence of a greater plaque stability in statin users [30].

This study has several limitations that should be highlighted. First, the retrospective design of the study does not permit to give a causal relevance to our findings. We just hypothesize potential associations that should be confirmed in prospective studies or randomized controlled trials. Second, causes of death cannot be established for most of patients so that an analysis of statin effect on specific events was not possible. Third, the dose of statins and baseline lipid levels, as well as new treatments and plasma lipid changes during follow-up were not available for most patients. Patients could have stopped or started new therapies during follow-up that could have influenced the mortality rate. Also, we were not able to estimate statin withdrawal associated with their adverse effects during the follow-up. Forth, this is a single center study with the experience of a hospital that serves the population of the North-East of Italy. In our center, as in others, CAS procedure is not the preferred procedure for significant carotid stenosis compared to surgical endarterectomy that results in the most prevalent choice. Therefore, our small sample number could have limited the evidence of the expected beneficial effect of statins on peri-procedural complications, although in the multivariate logistic analysis such an evidence was demonstrated (adjusted 6 times lower odds of developing peri-procedural complications with statin use, *p* = 0.007).

## 5. Conclusions

In this retrospective study we have demonstrated that baseline use of statin in patients that undergo CAS is associated with better long-term survival and lower rate of MACE occurrence than in patients not using statins. Among MACE, fatal and non-fatal stroke was the only event with a lower prevalence in statins-user patients at the end of the follow-up. Although this study does not provide a causal evidence, it provides the rationale for designing long-term prospective studies or randomized controlled trials to optimize drug therapy of patients who undergo CAS.

## Figures and Tables

**Table 1:** General characteristic of all studied patients and of patients divided by groups: censored or death, with or without new MACE occurrence, and using or not using statins at baseline.

Variable	All (*n* = 171)	Censored (*n* = 96)	Died (*n* = 75)	−MACE (*n* = 112)	+MACE (*n* = 59)	−Statins (*n* = 58)	+Statins (*n* = 113)
Enrolment age (years)	72 ± 8	70 ± 8	74 ± 9 ***	72 ± 9	72 ± 8	75 ± 8	70 ± 8 ***
Male sex (*n* (%))	125 (73)	70 (73)	55 (73)	86 (77)	39 (66)	43 (77)	82 (71)
Smoke history (*n* (%))	115 (67)	62 (64)	53 (71)	76 (68)	39 (66)	44 (76)	71 (63)
BMI (Kg/m^2^)	26.4 ± 3.8	26.3 ± 3.6	26.5 ± 4.1	26.2 ± 3.7	26.7 ± 4.0	25.8 ± 4.0	26.7 ± 3.7
SBP (mmHg)	159 ± 25	159 ± 23	159 ± 28	158 ± 24	161 ± 27	160 ± 28	158 ± 24
DBP (mmHg)	84 ± 13	82 ± 10	86 ± 17	84 ± 14	84 ± 12	85 ± 18	83 ± 11
eGFR (mL/min/1.73 m^2^)	67 ± 23	70 ± 21	64 ± 25	67 ± 23	67 ± 23	62 ± 22	70 ± 23 *
Hypertension (*n* (%))	146 (85)	77 (80)	69 (92)	96 (86)	50 (85)	46 (79)	100 (88)
Diabetes (*n* (%))	79 (46)	33 (34)	46 (61) ***	53 (47)	26 (44)	25 (43)	54 (48)
Dyslipidemia (*n* (%))	121 (71)	73 (76)	48 (64)	80 (71)	41 (69)	13 (22)	108 (96) ***
Atrial fibrillation (*n* (%))	16 (9)	4 (4)	12 (16) *	11 (10)	5 (8)	6 (10)	10 (9)
IHD (*n* (%))	61 (36)	34 (35)	27 (36)	37 (33)	24 (41)	12 (21)	49 (43) ***
Peripheral artery disease (*n* (%))	57 (33)	29 (30)	28 (37)	38 (34)	19 (32)	17 (29)	40 (35)
Carotid restenosis (*n* (%))	12 (7)	6 (6)	6 (8)	6 (5)	6 (10)	4 (7)	8 (7)
Preexistent MACE (*n* (%))	116 (68)	65 (68)	51 (68)	75 (67)	41 (69)	34 (59)	82 (73)
Statins users (*n* (%))	113 (66)	76 (79)	37 (49) ***	79 (70)	34 (58)	0 (0)	115 (100)
Antiplatelet users (*n* (%))	156 (91)	89 (93)	67 (89)	103 (92)	53 (90)	54 (93)	102 (90)
Follow-up (months)	87 ± 45	101 ± 44	69 ± 39 ***	86 ± 45	87 ± 45	74 ± 45	93 ± 43 **
New MACE (*n* (%))	59 (34)	35 (36)	24 (32)	0 (0)	59 (100)	25 (43)	34 (30)
Time to new MACE (months)	68 ± 50	78 ± 54	55 ± 42 **	86 ± 45	35 ± 42 ***	51 ± 47	77 ± 50 **
MACE within 1 month (*n* (%))	14 (8.2)	5 (5)	9 (12)	-	14 (100)	8 (14)	6 (5)
Deaths (*n* (%))	75 (44)	0 (0)	75 (100)	51 (45)	24 (41)	38 (65)	37 (33) ***
Time to death (months)	69 ± 39	-	69 ± 39	86 ± 45	89 ± 45	74 ± 45	93 ± 43 **

MACE, major adverse cardiovascular events; BMI, body mass index; SBP, systolic blood pressure; DBP, diastolic blood pressure; eGFR, estimated glomerular filtration rate; IHD, ischemic heart disease; −, without; +, with; * *p* < 0.050, ** *p* < 0.010, *** *p* < 0.001.

**Table 2:** Classic Cox proportional hazard regression analysis.

Variable	HR 95% CI	*p*
Enrolment age (years)	1.08 (1.04–1.13)	<0.001
Male sex (no/yes)	1.34 (0.70–2.55)	NS
Smoke history (no/yes)	0.77 (0.41–1.46)	NS
BMI (Kg/m^2^)	0.99 (0.91–1.09)	NS
eGFR (mL/min/1.73 m^2^)	1.00 (0.99–1.02)	NS
Hypertension (no/yes)	2.00 (0.83–4.81)	NS
Diabetes (no/yes)	2.45 (1.49–4.04)	<0.001
Preexistent MACE (no/yes)	0.96 (0.54–1.71)	NS
Statins users (no/yes)	0.56 (0.33–0.94)	0.028
Antiplatelet users (no/yes)	1.20 (0.57–2.55)	NS
Time to new MACE (months)	0.987 (0.981–0.992)	<0.001

BMI, body mass index; MACE, major adverse cardiovascular events; HR, hazard ratio; CI, confidence interval; NS, not significant; *p*, probability.

**Table 3:** Clock-forward multi-state Markov model analysis.

Variable		Transition 1 CAS to MACE (*n* = 59)		Transition 2 CAS to DEATH (*n* = 51)		Transition 3 MACE to DEATH (*n* = 24)	
		HR (95% CI)	*p*	HR (95% CI)	*p*	HR (95% CI)	*p*
Enrolment age (years)	<65	Ref.		Ref.		Ref.	
	65–75	1.43 (0.64–3.20)	NS	2.23 (0.68–7.27)	NS	0.04 (0.004–0.345)	0.004
	>75	1.42 (0.55–3.68)	NS	4.47 (1.23–16.10)	0.023	0.12 (0.02–0.84)	0.033
Sex	Female	Ref.		Ref.		Ref.	
	Male	0.52 (0.24–1.12)	NS	1.83 (0.70–4.77)	NS	1.78 (0.47–6.89)	NS
Smoke history	No	Ref.		Ref.		Ref.	
	Yes	1.12 (0.55–2.28)	NS	0.62 (0.28–1.36)	NS	0.40 (0.10–1.70)	NS
BMI class (Kg/m^2^)	<25	Ref		Ref.		Ref.	
	25–30	1.50 (0.81–2.81)	NS	1.68 (0.83–3.43)	NS	1.86 (0.55–6.32)	NS
	>30	1.08 (0.39–2.97)	NS	2.86 (0.97–8.37)	NS	3.50 (0.27–46.0)	NS
Stage of renal failure	Stage I	Ref.		Ref.		Ref.	
	Stage II	0.84 (0.37–1.90)	NS	0.54 (0.19–1.55)	NS	11.0 (0.74–162)	NS
	Stage III + IV	0.77 (0.28–2.09)	NS	2.13 (0.66–6.90)	NS	46.5 (2.09–1034)	0.015
Hypertension	No	Ref.		Ref.		Ref.	
	Yes	0.94 (0.43–2.06)	NS	1.60 (0.55–4.64)	NS	6.35 (0.43–93.9)	NS
Diabetes	No	Ref.		Ref.		Ref.	
	Yes	1.13 (0.66–1.94)	NS	2.29 (1.23–4.27)	0.009	5.00 (1.26–20.0)	0.022
Preexistent MACE	No	Ref.		Ref.		Ref.	
	Yes	1.64 (0.88–3.03)	NS	1.93 (0.96–3.89)	NS	0.31 (0.07–1.47)	NS
Statin users	No	Ref.		Ref.		Ref.	
	Yes	0.48 (0.27–0.85)	0.012	0.43 (0.22–0.84)	0.014	0.12 (0.03–0.47)	0.002
Antiplatelet users	No	Ref.		Ref.		Ref.	
	Yes	0.84 (0.35–1.98)	NS	1.37 (0.50–3.70)	NS	1.84 (0.42–8.14)	NS
New MACE occurrence	2.79 (0.01–556.4) NS						
Time to new MACE (months)	1.01 (0.98–1.03) NS						

CAS, carotid artery stenting; MACE, major adverse cardiovascular events; BMI, body mass index; Ref., reference group; HR, hazard ratio; CI, confidence interval; NS, not significant; *p*, probability.

**Table 4:** Description of class variables.

**Age Class (Years)**	**<65**	**65–75**	**>75**	***p***
*n* (%)	32 (19)	73 (43)	66 (38)	<0.001
Age	59 ± 2	70 ± 3	80 ± 4	
**BMI Class (Kg/m^2^)**	**<25**	**25–30**	**>30**	***p***
*n* (%)	71 (42)	76 (44)	24 (14)	<0.001
BMI	23.3 ± 1.5	27.2 ± 1.4	32.9 ± 4.1	
**Renal Failure Stage**	**I**	**II**	**III + IV**	***p***
*n* (%)	27 (16)	80 (47)	74 (37)	<0.001
eGFR (mL/min/1.73 m^2^)	124 ± 22	78 ± 11	47 ± 10	

BMI, body mass index; *p*, probability.

**Table 5:** Prevalence of newly occurred major cardiovascular events during follow-up in patients who used or did not use statins before carotid artery stenting.

Event	All Patients (*n* = 171)	−Statins (*n* = 58)	+Statins (*n* = 113)	*p*
Transitory ischemic attack (*n* (%))	26 (15)	9 (15)	17 (15)	NS
Fatal and non-fatal stroke (*n* (%))	23 (13)	13 (22)	10 (9)	0.018
Fatal and non-fatal ischemic heart disease (*n* (%))	35 (20)	10 (17)	25 (22)	NS

*p*, probability; NS, not statistically significant by Fisher exact test; −, without; +, with.
